# Extended phylogenetic analysis of a new Israeli isolate of *Brevicoryne brassicae virus* (BrBV-IL) suggests taxonomic revision of the genus *Iflavirus*

**DOI:** 10.1186/s12985-016-0500-z

**Published:** 2016-03-22

**Authors:** Neta Luria, Victoria Reingold, Oded Lachman, Noa Sela, Aviv Dombrovsky

**Affiliations:** Department of Plant Pathology, ARO, The Volcani Center, Bet Dagan, 50250 Israel

**Keywords:** *Iflavirus*, Aphid-infecting virus

## Abstract

**Background:**

*Brevicoryne brassicae virus* (BrBV) is a positive-strand genomic RNA virus which is unassigned tentative member of the genus *Iflavirus*. BrBv was first identified and characterized in the late 90’s in the cabbage aphid in the United Kingdom (UK) (J Gen Virol 88:2590–2595, 2007) and was fully sequenced, using random amplification of encapsidated RNA. No other reports have been published demonstrating detection of this virus outside the UK.

**Findings:**

A new isolate of the cabbage aphid virus *Brevicoryne brassicae virus* was identified from *Brevicoryne brassicae* aphids growing on wild mustard plants (*Sinapis arvensis*) in northern Israel. The virus genome was partially assembled from purified siRNA using the Illumina MiSeq Sequencing System with limited success. Combining classical viral RNA purification and RT-PCR amplification followed by traditional Sanger sequencing enabled obtaining the complete genomic sequence. The Israeli strain of BrBV shared 95 % nucleotide sequence identity with the BrBV found in the United Kingdom.

**Conclusions:**

The detection of BrBV in Israel indicates a broader geographical distribution of the virus”.

**Electronic supplementary material:**

The online version of this article (doi:10.1186/s12985-016-0500-z) contains supplementary material, which is available to authorized users.

## Background

In the 1990s, aphid-infecting viruses were discovered and characterized; most of them contained an RNA genome. The *Cripavirus* genus (family *Dicistroviridae*) includes two aphid viruses: *Rhopalosiphum padi virus* (RhPV) [1–3] and *Aphid lethal paralysis virus* (ALPV). ALPV was first isolated from the bird cherry-oat aphid, *Rhopalosiphum padi* [4, 5], then from the oleander aphid, *Aphis nerii* [6], and recently from the pea aphid, *Acyrthosiphon pisum* [7]. In addition, two unassigned aphid viruses have been reported in previous studies: *Acyrthosipon pisum virus* (APV) [2] and *Brevicoryne brassicae virus* (BrBV) [8]. Interestingly, BrBV was first discovered in the United Kingdom (UK) and was fully sequenced using random amplification of encapsidated RNA. The genome organization showed that the new virus belongs to the superfamily of Picorna-like viruses [8] No other reports have been published demonstrating detection of this virus outside the UK. Moreover, BrBV was detected in an aphid culture infested with aphid parasitoid which showed no increase in virus replication [8]. Currently, BrBV is considered an unassigned virus/tentative member of the genus *Iflavirus* [9], which includes nine species according to the 2014 report of the International Committee on Taxonomy of Viruses (ICTV) (http://www.ictvonline.org/virusTaxonomy.asp).

## Results

**Fig. 1:**
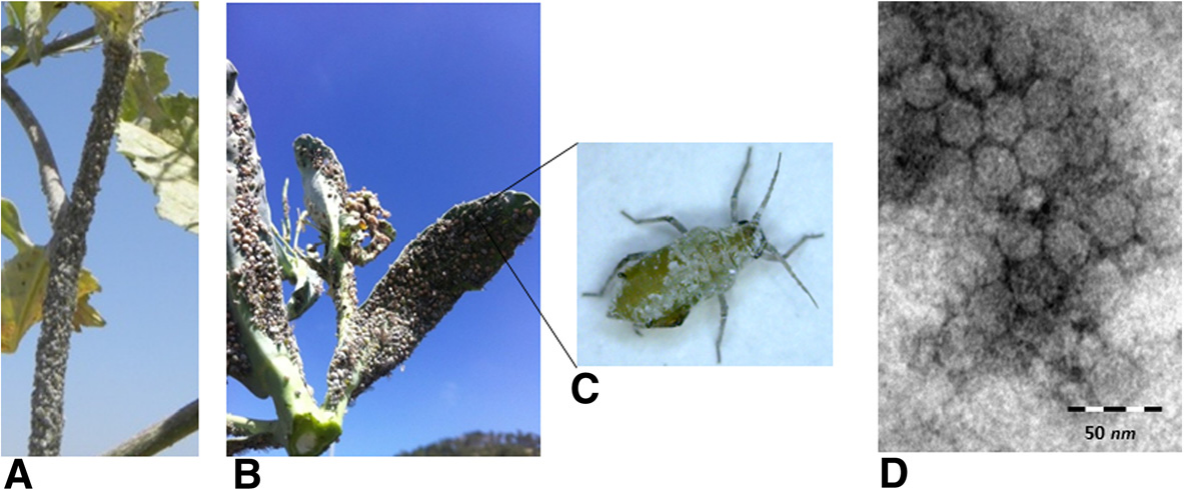
Characterization of the Israeli isolate of *Brevicoryne brassicae virus* (BrBV-IL). **a**–**b** Natural dense population of the cabbage aphid *B. brassicae* harboring BrBV-Is and infested by the parasitic wasp *Aphidius colemani* grown on wild mustard plants (*Sinapis arvensis*). **c** Microscope enlargement of adult *B. brassicae* aphid conaining the typical white waxes powder covered most of the body surface. **d** Electron micrograph of purified particles (*bar* represents 50 *nm*)

From 2009 to 2012, a survey aimed at identifying aphid-pathogenic viruses was conducted in Israel to find viruses that might be integrated into future biological aphid control. In the current study, BrBV-IL was found in a dense population of the cabbage aphid *Brevicoryne brassicae* that was highly infested by the parasitic wasp *Aphidius colemani* (Fig. 1a–c). The aphids were collected from wild mustard plants (*Sinapis arvensis*) growing in the northern coastal area of Israel and were further investigated in the current study. Later on, BrBV-IL was identified in two additional locations, Bet-Dagan and Modi’in, out of 12 *B. brassicae* samples of aphid populations across the country.

Viral RNA was purified from *B. brassicae* aphids using the Viral RNA Extraction Kit (Bioneer, Daejeon, South Korea) and served as the template for a reverse-transcription (RT) reaction carried out using Maxima Reverse Transcriptase (Fermentas–Thermo Fisher Scientific, Burlington, Canada) and the complementary primer R-5′-TTTAAAGAAAGAGCACTGTCCT-3′, followed by PCR amplification using JMR PCR mix (JMR Holdings, Kent, UK) and primer set no. 5 (Table 1), designed from the original BrBV genome (accession number NC_009530.1) [8]. Due to the high parasitoid wasp infestation of the original colony of *B. brassicae* aphids, individual adults were placed on cabbage leaves for 2 days, and then the larvae were collected and used to establish a new parasitoid-free colony of BrBV-IL-infected *B. brassicae* on *B. oleracea* plants. The established culture of *B. brassicae* aphids infected with BrBV-IL reared on brassica plants served for total RNA extraction using TRI reagent (Sigma, St. Louis, MO, USA), followed by mirVana miRNA Isolation Kit (Invitrogen, Carlsbad, CA, USA) for the enrichment of small RNA molecules. Recently, using NGS has become more and more advantageous for the discovery of new viruses and microorganisms. Moreover, the ability of whole viral assembly sequencing from small RNAs using rapid, accurate and high throughput technology allow to detect the presence of low concentration of new and cryptic viruses [10, 11].

**Table 1:** List of primers used for identification of the genome of the Israeli isolate of *Brevicoryne brassicae virus* (BrBV-IL) by RT-PCR amplification

Primer set no.	Amplicon position (nt.)	Forward primer (5′ → 3′)	Complementary primer (5′ → 3)
1	1–1962	TTGAAAAGATCGTGTAGGGTA	CCGGTTGTTACGGAAAGGTA
2	1895–3884	CCGTTCCATCCGATCTCTTA	CCGAATAAGTTTAACCCGTGTG
3	3653–5542	TGAAAGCTGTAATTAACTGTGTTGG	CAGGCTTACCATCAAGCCATA
4	5329–7128	AATGACATTCAGTGGCGTGA	AACGACAGGGCAGTCTTTTG
5	7088–8920	ATGCACCTTGGTGTGCCTAT	GAGCTAGTTCCGACCACTCG
6	8300–10166	TAACTCCTCGCGACCCTAGA	TTTAAAGAAAGAGCACTGTCCT

**Fig. 2:**
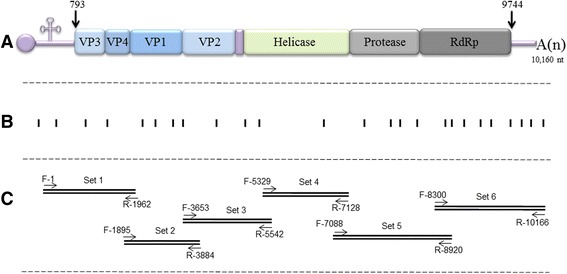
Schematic representation of *Brevicoryne brassicae virus* (BrBV-IL) genome and illustration of the sequencing strategy. **a** Genomic structure of BrBV-IL genome, including the viral protein genome-linked (VPg), 5′UTR (792 nt) and 3′UTR including the 3′ polyadenylated tail (419 nt including the stop codon). The long polyprotein encodes non-structural proteins, including motifs of chymotripsin-like proteinase, NTPase/helicase and RNA-dependent RNA polymerase (RdRp), and of viral structural proteins VP1–VP4. **b** The strategy used for BrBV-IL genome sequencing. The *rectangles* below the genome map represent the locations and lengths of sequences obtained by NGS analysis of siRNA. **c** The sequenced clones obtained by RT-PCR amplification using six primer sets (Table 1) to map the entire viral genome

The small RNA was used for the construction of a miRNA library using TruSeq Small RNA Sample Preparation Kit (Illumina, San Diego, CA, USA). The library was sequenced by the Illumina MiSeq Sequencing System (Volcani Center) and provided 217,324 reads (with 26,490 unique reads), of which only 46 reads matched the BrBV genome (accession number NC_009530.1) (Fig. 2, b).

The low genome coverage obtained with the next generation sequencing (NGS) analysis forced us to combine the traditional sequencing method based on RT-PCR amplification to retrieve the complete genome sequence.

Viral purification was carried out on 100 g brassica leaves carrying a dense population of BrBV-IL-infected *B. brassicae* [6]. The presence of viral particles (Fig. 1d) was verified by transmission electron microscopy (Tecnai G2, FEI-Philips, Eindhoven, The Netherlands). Viral RNA was then extracted using the Viral RNA Extraction Kit and served as a template for a RT reaction, in which oligo dT(17)VN, random hexamer and BrBV-specific primer (5′-TTTAAAGAAAGAGCACTGTCCT-3′) were combined. The obtained cDNA was used as a template for the PCRs using six pairs of specific primers (Table 1) (Fig. 2c) which were designed mostly according to the BrBV genome from GenBank (accession number NC_009530.1) [8] and the obtained reads from the NGS data. The amplicons were cloned into pGEM-T-easy vector (Promega, Madison, WI, USA) and 2–4 clones harboring the overlapped amplicons were sequenced from both directions (HyLabs, Rehovot, Israel). The complete genome sequence of the Israeli strain of BrBV (BrBV-IL), comprised of 10,160 nt (excluding the 3′ polyadenylated terminal tail) (Fig. 2a) encoding a putative long open reading frame (ORF) with 2983 amino acids and two untranslated regions (UTRs) at the 5′ (792 nt) and 3′ (419 nt including the stop codon) ends, was submitted to GenBank under accession number KP777548.

Sequence homology of BrBV-IL determined using the Basic Local Alignment Search Tool (BLAST; http://blast.ncbi.nlm.nih.gov/Blast.cgi) revealed 95 % nucleotide sequence identity and 98 % deduced amino acid sequence identity to the UK isolate of BrBV (accession number NC_009530.1).

**Fig. 3:**
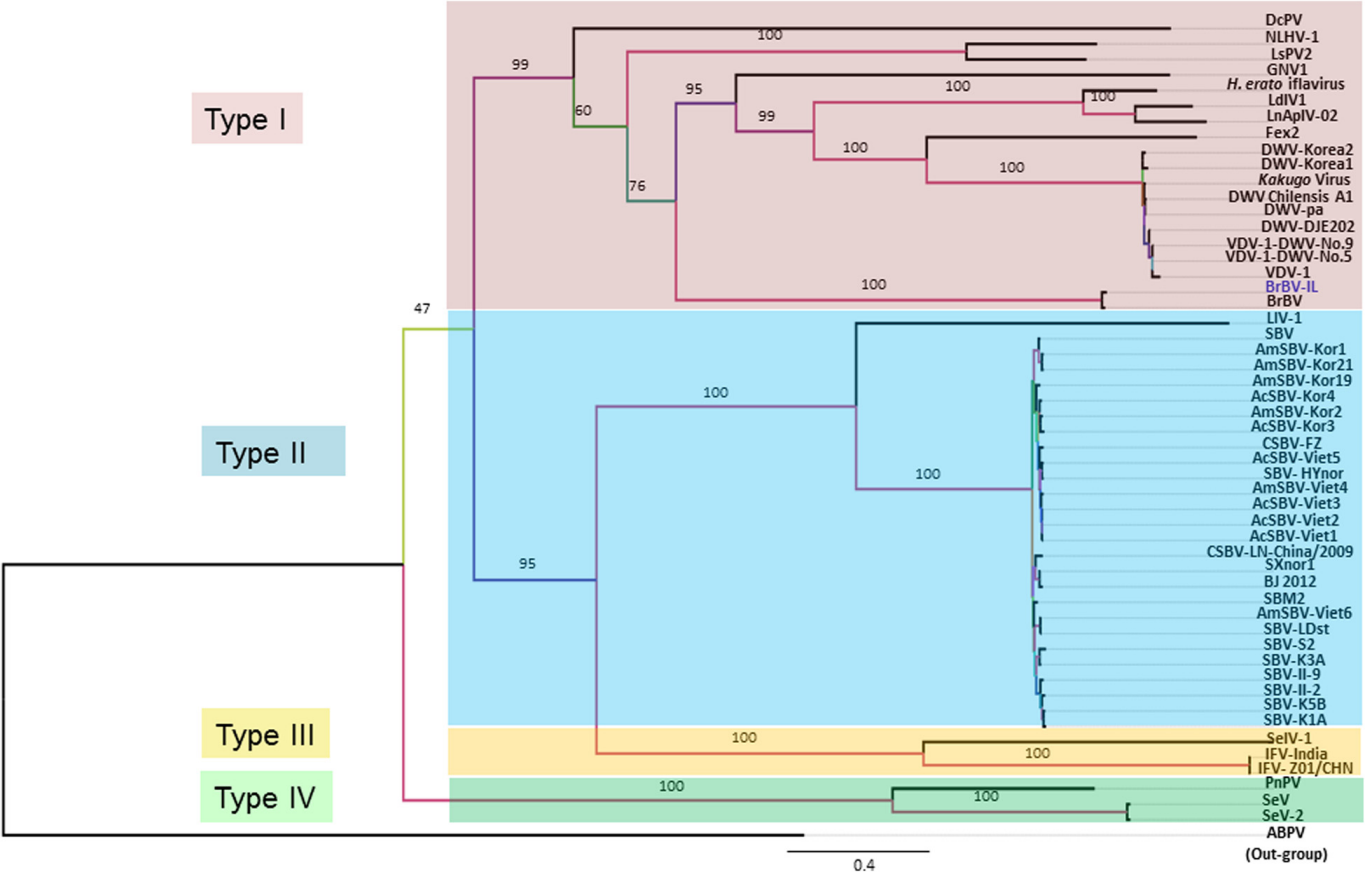
Phylogenetic analysis of *Brevicoryne brassicae virus* (BrBV). Rooted phylogenetic tree based on the deduced amino acid sequences of the polyproteins of some members of the genus *Iflavirus*. Bootstrap values of 100 replicates are indicated. For visualization we used FigTree sofrtware [17] Viral sequences (with abbreviations and NCBI/GenBank accession numbers): *Heliconius erato iflavirus* (*H.erato* iflavirus; KJ679438), *Spodoptera exigua iflavirus 2* isolate Korean (SeV; JN870848), *Deformed wing virus* isolate Chilensis A1 (DWV Chilensis A1; JQ413340), *Lymantria dispar iflavirus 1* isolate Ames (LdiV1; KJ629170), *Antheraea pernyi iflavirus* isolate LnApIV-02 (LnApIV-02; KF751885), *Perina nuda picorna-like virus* (PnPV; AF323747), *Sacbrood virus* strain AcSBV-Kor4 (AcSBV-Kor4; KP296803)*, Sacbrood virus* strain AcSBV-Kor3 (AcSBV-Kor3; KP296802), *Sacbrood virus* strain AmSBV-Kor2 (AmSBV-Kor2; KP296801), *Sacbrood virus* strain AmSBV-Kor1 (AmSBV-Kor1; KP296800), *Sacbrood virus* isolate AmSBV-Viet6 (AmSBV-Viet6; KM884995), *Sacbrood virus* isolate AcSBV-Viet5 (AcSBV-Viet5; KM884994), *Sacbrood virus* isolate AmSBV-Viet4 (AmSBV-Viet4; KM884993), *Sacbrood virus* isolate AcSBV-Viet3 (AcSBV-Viet3; KM884992), *Sacbrood virus* isolate AcSBV-Viet2 (AcSBV-Viet2; KM884991), *Sacbrood virus* isolate AcSBV-Viet1 (AcSBV-Viet1; KM884990), *Sacbrood virus* isolate CSBV-FZ (CSBV-FZ; KM495267), *Spodoptera exigua iflavirus 2* (SeV-2; KJ186788), *Sacbrood virus* strain SBM2 (SBM2; KC007374), *Lygus lineolaris virus 1* isolate LlV-1 (LlV-1; JF720348), *Sacbrood virus* strain AmSBV-Kor19 (AmSBV-Kor19; JQ390592), *Sacbrood virus* strain AmSBV-Kor21 (AmSBV-Kor21; JQ390591), *Varroa destructor virus 1* (VDV-1; AY251269), *Infectious flacherie virus* strain IFV-India (IFV-India; HM569717), *Nilaparvata lugens honeydew virus-1* (NLHV-1;AB766259), *Brevicoryne brassicae picorna-like virus* isolate IL (BrBV-IL; KP777548), *Sacbrood virus* CSBV-LN/China/2009 (CSBV-LN/China/2009; HM237361), *Dinocampus coccinellae paralysis virus* strain Quebec2013 (DcPV;KF843822), *Deformed wing virus* isolate Varroa-infested-colony-DJE202 (DWV- DJE202; KJ437447), *Sacbrood virus* isolate SXnor1 (SXnor1; KJ000692), *Sacbrood virus* strain BJ 2012 (BJ 2012; KF960044), *Deformed wing virus* strain Korea-2 (DWV-Korea2; JX878305), *Deformed wing virus* strain Korea-1 (DWV-Korea1; JX878304), *Formica exsecta virus 2* isolate Fex2 (Fex2; KF500002), *Sacbrood virus* strain II-9 (SBV-II-9; JX270800), *Sacbrood virus* strain S2 (SBV-S2; JX270799), *Sacbrood virus* strain K3A (SBV-K3A; JX270798), *Sacbrood virus* strain K5B (SBV-K5B; JX270797), *Sacbrood virus* strain K1A (SBV-K1A; JX270796), *Sacbrood virus* strain II-2 (SBV-II-2; JX270795), *Deformed wing virus* isolate VDV-1-DWV-No-9 (VDV-1-DWV-No-9; HM067438), *Deformed wing virus* isolate VDV-1-DWV-No-5 (VDV-1-DWV-No-5; HM067437), *Deformed wing virus* isolate PA (DWV-pa; AY292384), *Infectious flacherie virus* strain ZheJiang01/CHN (IFV-Z01/CHN; EU868609), *Brevicoryne brassicae picorna-like virus* (BrBV; EF517277), *Sacbrood virus* (SBV; AF092924), *Kakugo virus* (*Kakugo virus*; AB070959), *Spodoptera exigua Iflavirus-1* (SelV-1; JN091707), *Laodelphax striatellus picorna-like virus 2* isolate LsPV2 (LsPV2; KM272628), *Graminella nigrifrons virus 1* isolate Ohio (GNV1; KP866792), *Sacbrood virus* strain HYnor (SBV-HYnor; KJ959614) and *Sacbrood virus* strain LDst (SBV-LDst; KJ959613). *Acute bee paralysis virus* (ABPV; NC_002548) served as outgroup

Multiple sequence alignments were analyzed using MAFFT [12] software programs. Phylogenetic trees predictions were carried out using phyml with both the long ORF amino acid sequences (Fig. 3 and Additional file 1: Figure S1-A) as well as RdRp amino acid sequence only (Additional file 1: Figure S1-B). First, the MAFFT program was used to align each ORF [12]. Then, a phylogenetic tree was constructed based on a maximum likelihood (ML) framework, using PhyML software [13] with 100 bootstrap replicates [14]. For the analysis RdRp tree we defined the RdRp region by using the NCBI conserved domain server [15]. To further confirm our sub-taxonomic groups definition analysis we also constructed a Bayesian MCMC phylogenetic tree based on the full nucleotide sequences of the viruses using MrBayes software [16] (Additional file 1: Figure S1-C). This tree also agreed on the sub-clsification determined by the previous phylogenetic trees.

Based on our NGS analysis of siRNA purified from aphids, we obtained reads matching the BrBV-IL genome. However, only preliminary knowledge of the potential infecting virus in *B. brassicae* allowed identification of BrBV-IL, as the reads were not mapped onto significant contigs. The possibility of resequencing (assembling the reads based on the UK isolate of the BrBV genome) enabled locating the 46 reads (0.0002 % of the reads, covering 11.5 % of the genome) within the genome, enabling a first determination of the viral agent with only preliminary information on nucleotide sequence. The complete genome sequence of BrBV-IL was obtained by traditional RT-PCR amplification followed by nucleotide sequencing (Fig. 2c). Although the taxonomic affiliation of BrBV is unclear, this study is the second report on this virus, and as such provides evidence for its global spread in *B. brassicae* populations.

Based on a rooted phylogenetic tree analysis of the long ORF encoding the polyprotein of the viruses belonging to the *Iflavirus* genus, the two unassigned BrBV isolates and an outgroup-*Acute bee paralysis virus* (NC_002548) in the family *Dicistroviridae*, genus *Aparavirus* (Fig. 3), it is suggested that the *Iflavirus* genus be divided into four tentative clusters: type I containing the type member *Infectious flacherie virus* (IFV), type II and type III (Fig. 3). As shown in Fig. 3, the two BrBV isolates are located in the type II area of the phylogenetic tree, clustering together on a separate branch.

Future discovery of new members of the family *Iflaviridae* and further studies should shed additional light on the taxonomic affiliation among the genera. In addition, pathogenicity experiments aimed at testing the infectivity potential of BrBV-IL on different aphid species could potentially lead to future application as a biopesticide.

### Availability of supporting data

BrBV-IL genome sequence was submitted to genbank under accession number KP777548.

## Additional file

Additional file 1: Figure S1. Four sub-taxomonic groups were classified according to three different trees constructed with different sequences and methodologies. (DOCX 421 kb)

## Abbreviations

BrBv: Brevicoryne brassicae virus
BrBV-IL: Israeli isolate of *Brevicoryne brassicae virus*
PCR: polymerase chain reaction
NGS: next generation sequencing
sRNA: small RNA
ORF: open reading frame
