# Supplementary material for: Extended phylogenetic analysis of a new Israeli isolate of Brevicoryne brassicae virus (BrBV-IL) suggests taxonomic revision of the genus Iflavirus
Source: Virol J. 2016 Mar 22;13:50. doi: 10.1186/s12985-016-0500-z (PMC4802879; doi:10.1186/s12985-016-0500-z)

**Additional file 1: Figure S1**: four sub-taxomonic groups were classified according to three different trees constructed with different sequences and methodologies: (A) phylogentic tree of polyprotein constructed with phyml using LG amino acid model with 1000 bootstrap (B) phylogentic tree of RdRp protein constructed with phyml using LG amino acid model with 1000 bootstrap (C) phylogentic tree of the viral capsid sequence. The sequences were aligned using mafft after wards Gblocks was used to correct the alignment and then a tree was constructed with phyml using LG model with 100 bootstrap (D) Bayesian phylogenetic analysis of whole nucleotide sequences of the virus using MrBayes software with the parameters lset nst=6 rates=invgamma mcmc ngen=300000 of the following viral sequences: Heliconius erato iflavirus (KJ679438), Spodoptera exigua iflavirus 2 isolate Korean (JN870848), Deformed wing virus isolate Chilensis A1 (JQ413340), Lymantria dispar iflavirus 1 isolate Ames (KJ629170), Antheraea pernyi iflavirus isolate LnApIV-02 (KF751885), Perina nuda picorna-like virus (AF323747), Sacbrood virus strain AcSBV-Kor4 (KP296803), Sacbrood virus strain AcSBV-Kor3 (KP296802), Sacbrood virus strain AmSBV-Kor2 (KP296801), Sacbrood virus strain AmSBV-Kor1 (KP296800), Sacbrood virus isolate AmSBV-Viet6 (KM884995), Sacbrood virus isolate AcSBV-Viet5 (KM884994), Sacbrood virus isolate AmSBV-Viet4 (KM884993), Sacbrood virus isolate AcSBV-Viet3 (KM884992), Sacbrood virus isolate AcSBV-Viet2 (KM884991), Sacbrood virus isolate AcSBV-Viet1 (KM884990), Sacbrood virus isolate CSBV-FZ (KM495267), Spodoptera exigua iflavirus 2 (KJ186788), Sacbrood virus strain SBM2 (KC007374), Lygus lineolaris virus 1 isolate LlV-1 (JF720348), Sacbrood virus strain AmSBV-Kor19 (JQ390592), Sacbrood virus strain AmSBV-Kor21 (JQ390591), Varroa destructor virus 1 (AY251269), Infectious flacherie virus strain IFV-India (HM569717), Nilaparvata lugens honeydew virus-1 (AB766259), Brevicoryne brassicae picorna-like virus isolate IL (KP777548), Sacbrood virus CSBV-LN/China/2009 (HM237361), Dinocampus coccinellae paralysis virus strain Quebec2013 (KF843822), Deformed wing virus isolate Varroa-infested-colony-DJE202 (KJ437447), Sacbrood virus isolate SXnor1 (KJ000692), Sacbrood virus strain BJ 2012 (KF960044), Deformed wing virus strain Korea-2 (JX878305), Deformed wing virus strain Korea-1 (JX878304), Formica exsecta virus 2 isolate Fex2 (KF500002), Sacbrood virus strain II-9 (JX270800), Sacbrood virus strain S2 (JX270799), Sacbrood virus strain K3A (JX270798), Sacbrood virus strain K5B (JX270797), Sacbrood virus strain K1A (JX270796), Sacbrood virus strain II-2 (JX270795), Deformed wing virus isolate VDV-1-DWV-No-9 (HM067438), Deformed wing virus isolate VDV-1-DWV-No-5 (HM067437), Deformed wing virus isolate PA (AY292384), Infectious flacherie virus strain ZheJiang01/CHN (EU868609), Brevicoryne brassicae picorna-like virus (EF517277), Sacbrood virus (AF092924), Kakugo virus (AB070959), Spodoptera exigua Iflavirus-1 (JN091707), Laodelphax striatellus picorna-like virus 2 isolate LsPV2 (KM272628), Graminella nigrifrons virus 1 isolate Ohio (KP866792), Sacbrood virus strain HYnor (KJ959614) and Sacbrood virus strain LDst (KJ959613). Acute bee paralysis virus (NC_002548) served as the outgroup. The four different groups are marked in Latin numbers. It can be seen that all three trees agrees on the sub-taxonomic compartment.


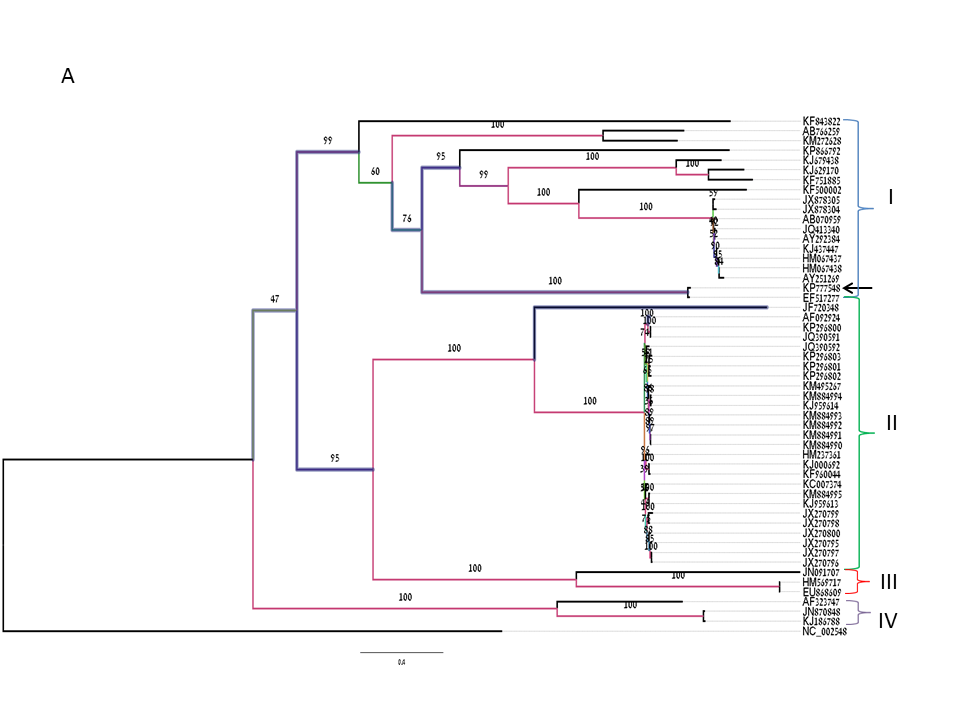


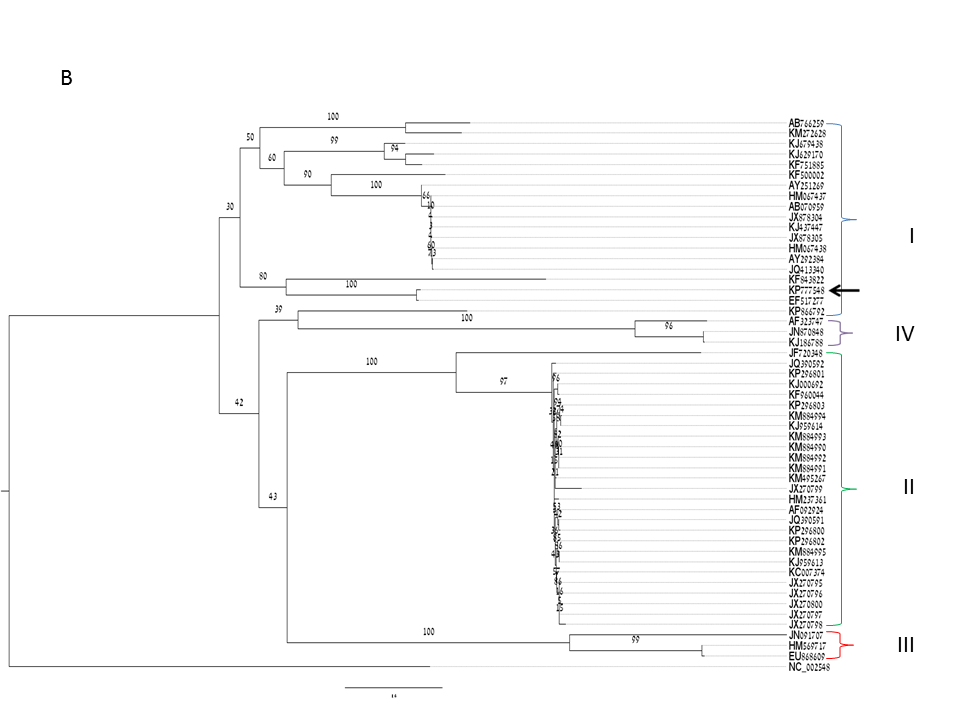


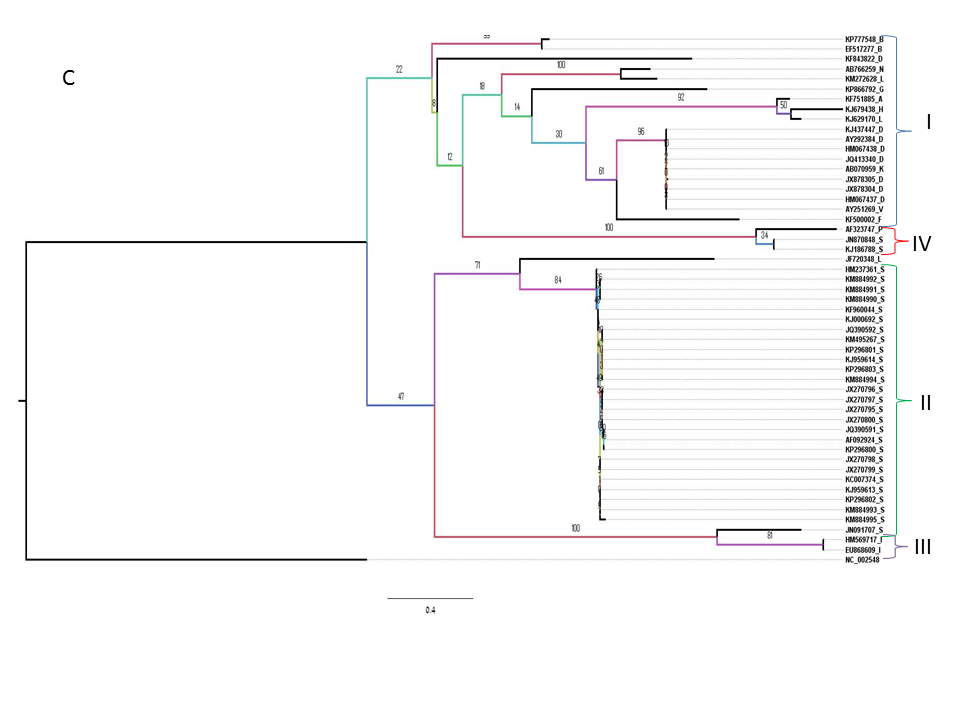


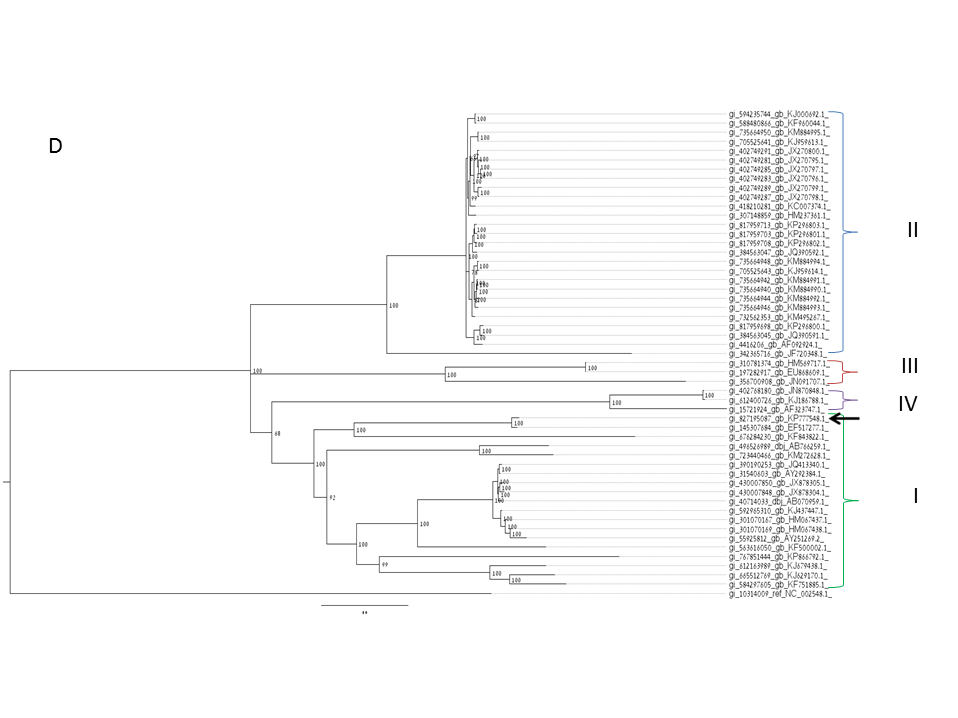

Supplement: Additional file 1: Figure S1. — Four sub-taxomonic groups were classified according to three different trees constructed with different sequences and methodologies. (DOCX 421 kb) [file 12985_2016_500_MOESM1_ESM.docx]
